# Mutation in *MPT64* gene influencing diagnostic accuracy of SD Bioline assay (capilia)

**DOI:** 10.1186/s12879-019-4671-2

**Published:** 2019-12-11

**Authors:** Kamal Singh, Richa Kumari, Rajneesh Tripathi, Ankush Gupta, Shampa Anupurba

**Affiliations:** 1 Department of Microbiology, Institute of Medical Sciences, Banaras Hindu University, Varanasi, Uttar Pradesh India; 2 Department of Biochemistry, Institute of Science, Banaras Hindu University, Varanasi, Uttar Pradesh India

**Keywords:** MPT64, MOTT, Capilia, MTBDR *plus* assay, Mutation, *Mycobacterium tuberculosis* complex (MTBC)

## Abstract

**Background:**

Success of India’s TB control program relies on rapid case detection, monitoring, care and treatment of drug resistance. Patients on multidrug resistance (MDR) treatment are monitored by follow up cultures. Discordant results (culture and smear positive while capilia negative) are usually declared negative *Mycobacterium tuberculosis* complex (MTBC). This study was designed to understand the possible causes of discordant results.

**Methods:**

The capilia kit was evaluated to test its utility among 4737 follow up MDR patients enrolled during a period of 1 year. A total of 889 were liquid culture positive, 3375 were negative and 473 were contaminated. Of the 889 cultures positive, 829 were found positive by ZN smear, capilia test and MTBDR *plus* assay. The cultures which gave a positive result on Mycobacterium Growth Indicator Tube 960 (MGIT 960) and ZN smear but were negative on capilia test with no growth on Brain Heart Infusion agar (BHI) were included in this study. The conflicting results of capilia were compared with other molecular techniques; MTBDR *plus* assay and DNA sequence analysis of *MPT64* gene.

**Results:**

Out of 889 culture positive, 60 (6.7%) were found positive on liquid culture and ZN smear but were negative on capilia. Of these 60 cultures, 10 (16.7%) were found positive by both MTBDR *plus* assay and PCR. The sequencing analysis revealed that all of the capilia negative isolates had mutations within the *MPT64* gene.

**Conclusion:**

Re-evaluation of culture positive but capilia negative isolates should be done before declaring them as Mycobacterium other than tuberculosis (MOTT) because such cases can act as chronic carriers of TB in the population which can lead to the rise of this lethal disease.

## Background

Tuberculosis (TB) is a disease since ancient times which still remains a major public health challenge and one of the top causes of death in the twenty-first century with a mortality of 1.4 million cases globally and 0.48 million cases in India [1]. The global project on drug resistance surveillance provides a standardized overview of the prevalence of drug resistance in many countries across the world [2]. Despite several technological advancements in the diagnostics and susceptibility testing, still there are some loopholes. The developing countries are facing many problems regarding the diagnosis of this disease such as limited resources, delay in diagnosis and lack of education [3].

.According to World Health Organization (WHO), all the drug resistant cases are primarily diagnosed by rapid tools such as GeneXpert or MTBDR *plus* assay (also known as line probe assay). After diagnosis of MDR-TB (multidrug resistant) the patients are put on drug resistant treatment and the success of treatment is being monitored by culture follow up [4]. However, even with a positive liquid culture there is a need to differentiate *Mycobacterium tuberculosis* complex (MTBC) from Mycobacterium other than tuberculosis (MOTT) bacilli. This can be easily achieved by the currently available molecular methods but these techniques require good infrastructure and skilled human resources [5, 6]. Thus a rapid, sensitive and simple test for quick identification of the MTBC is necessary for accurate diagnosis and proper treatment of the disease.

Recently, rapid methods of identifying MTBC from the Acid-fast bacilli (AFB) smear-positive cultures are possible by TB-Neo assay (Tauns Laboratories, Inc., Numazu, Japan) and the SD Bioline TB Ag MPT64 assay (capilia) (Standard Diagnostics, Yongin-si, Gyeonggi-do, Republic of Korea). These assays have the advantage of being inexpensive, easy to use and readily available, even in low-resource settings [7]. They are easily stored at room temperature and allow results from positive cultures within 15 min [8].Capilia is an MPT64 based, simple and rapid immuno-chromatographic identification test for the MTBC that uses mouse monoclonal anti-MPT64. The MPT64 is one of the major culture filtrate protein (24 kDa) encoded by the RD2 region gene [9]. However some discrepancies in capilia have been reported in previous studies [&, 10–16]. Follow up cultures from TB patients diagnosed to harbor resistant MTBC and started on MDR treatment were found positive in culture and microscopy but negative by capilia. In this regard the study was designed to elucidate the reason for such discordant results in capilia by comparing with other standard testing methods like MTBDR *plus* assay and DNA sequencing.

## Methods

### Study design and specimens

This was a cross-sectional study conducted in the Department of Microbiology, Institute of Medical Sciences, Banaras Hindu University, Varanasi. Present study is a part of routine diagnostic workflow under RNTCP (Revised National Tuberculosis Control Program). The collected specimens were first subjected to GeneXpert which detected rifampicin resistance (RR). Since RR is a surrogate marker for MDR TB, diagnosis of MDR TB was based on GeneXpert only. Routine cultures were performed to monitor the response to MDR TB treatment. During 1 year follow up of MDR patients (total 4737) 889 were liquid culture positive, 3375 were negative and 473 were contaminated. Of the 889 cultures positive, 829 were found positive by ZN smear, capilia test and MTBDR *plus* assay. The cultures which gave a positive result on MGIT 960 and ZN smear but were negative on capilia test with no growth on Brain Heart Infusion (BHI) agar were included in this study. Further evaluation of negative capilia results was done in this study.

### Specimen processing and culture

The collected specimens were decontaminated as described elsewhere [17, 18]. Briefly, specimens were decontaminated using N-acetyl-L-cysteine and sodium hydroxide (NALC-NaOH) method. The decontaminated sediments were inoculated into BD BACTEC MGIT 960 automated liquid culture system, until the instrument gave positive result or kept for 6 weeks.

### Identification of positive cultures by capilia

All the cultures that were positive by MGIT 960 were subjected to direct ZN smear microscopy and capilia.

### Genomic DNA extraction

DNA extraction was performed in the BSL-3 laboratory from the AFB positive culture as previously described with some modifications [19, 20]. In brief, CTAB-chloroform method was used for DNA extraction from 1 mL of MGIT culture. The quality and quantity of DNA were analyzed with the help of spectrophotometer (Thermo Scientific NanoDrop 2000).

### GenoType MTBDR *plus* assay

The GenoType MTBDR *plus* (line probe assay) was performed in three separate rooms, according to the manufacturer’s (HainLife science, Nehren, Germany) instructions. There are three steps for MTBDR *plus* assay, first DNA extraction from culture isolates, second amplification of rifampicin resistance determining region (RRDR) region by Multiplex PCR and reverse hybridization. Multiplex PCR was performed using a 45 μl amplification mix consisting of 10 μl AM- A and 35 μl AM-B. The 5 μl DNA template was added to each tube in a separate room and amplification was performed with the final volume of 50 μl using a thermal cycler and amplification protocol provided by Hain Lifescience. Reverse hybridization was performed in TwinCubator as per manufacturer’s instructions. After completion of hybridization, the strips were washed, removed and fixed to GenoType MTBDR *plus* assay worksheet for interpretation [21].

### PCR for *MPT64* gene

Primers for PCR were designed in house to amplify *MPT64* gene from flanking region (Table 1). Reaction mixture was prepared containing 2.5 μl of 10X reaction buffer (GeNei, Bangalore, India), 2 μl of 200 M concentrations of each of the deoxynucleoside triphosphates (dNTPs) (GeNei, Bangalore, India), 0.3 μl of 5 U Taq DNA Polymerase (GeNei, Bangalore, India), 1 μl of the each oligonucleotide primers MPTF and MPTR (10 pmol each) (GeNei, Bangalore, India), 5 μl (50 ng) of the DNA template and milli Q to maintain the final volume of 25 μl. The following reaction conditions was used for amplification: initial denaturation at 95 °C for 5 min, 30 cycles of 95 °C for 30 s, 59 °C for 45 s, 72 °C for 45 s and a final elongation step at 72 °C for 10 min. The H37Rv was used as positive control and PCR grade water was used as a negative control.

**Table 1:** Oligonucleotide used as primer for amplification of *MPT64* gene PCR

Gene	Primer sequence(5–3)	Product size	Reference
MPT64 forward (MTPF)	ACCGAACACTCATTTCCGC	771 bp	In this study
MPT64 reverse (MTPR)	CTACTCCCGGAGGAATTTCG	771 bp	In this study

### Sequencing of *MPT64* gene

For sequencing the MPT64 region was amplified with the help of MPTF and MPTR primers. Product size was confirmed by agarose (2%) gel electrophoresis and sent for sequencing.

### Sequence data analysis

The sequence of 1 control (H37Rv) and 10 mutants obtained were analyzed by using BioEdit version 7.0.5.3 software tool. All the mutant sequences were compared with the control (H37Rv) sequence by using Clustal W multiple sequence alignment on BioEdit software and the mutations in the nucleotide sequences were marked. After nucleotide sequence analysis both the control (H37rv) and mutant nucleotide sequences were in-vitro translated on ExPASY translate. The in-vitro translated sequences of both control (H37Rv) and mutant proteins were also analyzed by Clustal W multiple sequence alignment on BioEdit software and also the mutations in the protein sequences were marked.

### Statistical analysis

The sensitivity and specificity of capilia was calculated using 2018 MedCalc software (version: bvba). GenoType MTBDR *plus* Assay was taken as gold standard.

## Results

**Fig. 1:**
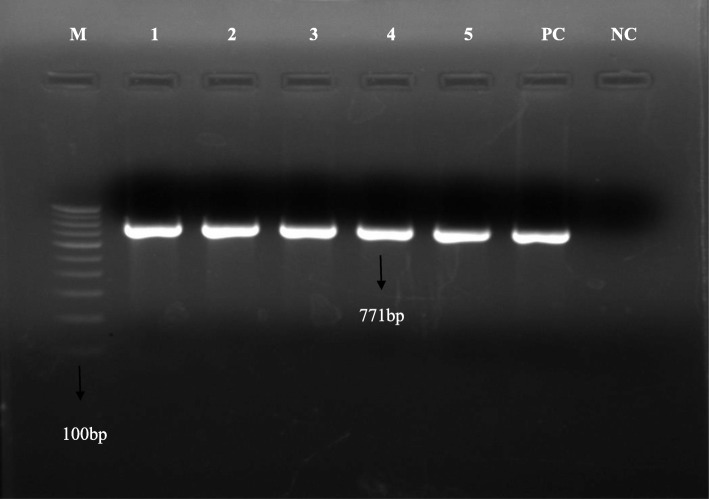
Amplified PCR product of *MPT64* gene. M: Marker 100 bp; Lane1–5: positive band for *Mycobacterium.tuberculosis* (*MPT64* gene); PC: Positive control (H37Rv); NC: Negative control (PCR grade water)

In this study, there were a total of sixty follow up cases from whom the specimens were positive on liquid culture and ZN smear but negative by capilia test. Of these 60 cases, 38 (63.3%) were males and 22 (36.7%) were females. Mean age of the study subjects was 27.6 ± 7.9 years. 35 (58.3%) patients were in the intensive phase of MDR treatment, whereas remaining 25 (41.7%) were in the continuation phase. Out of 60 capilia negative cultures, 10 (16.7%) were found positive by MTBDR *plus* assay and PCR (Fig. 1). The remaining 50 cultures that were negative by both capilia and MTBDR *plus* assay were probably MOTT.

In order to identify and characterize the discrepancies in the results of capilia, 10 positive cultures and 1 control (H37Rv) were analyzed by sequencing *MPT64* gene. In our study, the sequence analysis revealed that three major positions of the *MPT64* gene had mutated by either deletion or insertion of nucleotide. In 40% of the mutant positive cultures (K1, K3, K6 and K9) deletion of G nucleotide was found at 26th position in the *MPT64* gene, leading to frameshift mutation from 9th amino acid until a stop codon occurred after 41st amino acid. Interestingly, in another 40% of the mutant positive cultures (K2, K4, K5 and K8) deletion of C nucleotide at 37th position in the *MPT64* gene was present, leading to frameshift mutation from 13th amino acid until a stop codon occurred either after 41st amino acid in 3 mutant positive culture (K2, K5 and K8) or after 37th amino acid in 1 mutant positive culture (K4). However, in the remaining 20% mutant positive cultures(K7 and K10) there was an insertion of G nucleotide at the 6th position leading to a frameshift mutation from 3^rd^amino acid until a stop codon occurred after 88th amino acid in 7th positive culture(Table 2). The sensitivity, specificity, positive predictive and negative predictive values of the capilia test (95% CI) were found to be 98.81% (97.82 to 99.43%), 100% (92.89 to 100%), 100 and 83.33% (72.97 to 90.25%) respectively (Table 3).

**Table 2:** Detail illustration of mutations in *MPT64* gene and their effects in capilia negative cultures

Sample Number	Liquid Culture	ZN Smear	Capilia	MTBDR *plus* assay	Mutation	Phenotypic effect
K1	+	+	–	+	26th position G deletion	Frameshift from 9th amino acid leading to stop codon after 41st amino acid
K2	+	+	–	+	37th position C deletion	Frameshift from 13th amino acid leading to stop codon after 41st amino acid
K3	+	+	–	+	26th position G deletion	Frameshift from 9th amino acid leading to stop codon after 41st amino acid
K4	+	+	–	+	37th position C deletion	Frameshift from 13th amino acid leading to stop codon after 37th amino acid
K5	+	+	–	+	37th position C deletion	Frameshift from 13th amino acid leading to stop codon after 41st amino acid
K6	+	+	–	+	26th position G deletion	Frameshift from 9th amino acid leading to stop codon after 41st amino acid
K7	+	+	–	+	6th position G insertion	Frameshift from 3rd amino acid leading to stop codon after 88th amino acid
K8	+	+	–	+	37th position C deletion	Frameshift from 13th amino acid leading to stop codon after 41st amino acid
K9	+	+	–	+	26th position G deletion	Frameshift from 9th amino acid leading to stop codon after 41st amino acid
K10	+	+	–	+	6th position G insertion	Frameshift from 3rd amino acid leading to stop codon after 88th amino acid
K11 (Control)	+	+	+	+	No mutation

**Table 3:** Performance of capilia test as compared to MTBDR *plus* assay

MTBDR *plus* assay	Capilia test				95% CI
	Positive	Negative	Total	Sensitivity = 98.81	97.82 to 99.43%
Positive	829	10	839	Specificity = 100	92.89 to 100%
Negative	0	50	50	PPV = 100	–
Total	829	60	889	NPV = 83.33	72.97 to 90.25%

## Discussion

Despite recent advances in tuberculosis research, its diagnosis still remains a global challenge. Nowadays rapid identification and diagnosis of MTBC is based on MTBDR *plus* assay, GeneXpert and culture followed by capilia. Although these techniques are sensitive, still there are some loopholes**.** MTBDR *plus* assay has been developed for the detection of MTBC as well as resistance to rifampicin and isoniazid (RIF and INH). However, it requires dedicated equipment and technical expertise. In recent times MTBC from MGIT positive cultures are detected with the help of rapid immuno-chromatographic tests like TB-Neo assay and SD Bioline TB Ag MPT64 assay (capilia). However, there are a few studies showing that capilia test can give false negative result [&, 11–16]. A previous study reported that MPT64 used in SD Bioline TB Ag MPT64 assay (capilia) and the gene encoding MPB64 (Capilia TB-Neo assay) to be identical except for one silent mutation [22]. In present study we found such cases which were capilia negative. We compared capilia results with microscopy, MTBDR *plus* assay and PCR.

In this study we got 10 (16.67, 95% CI 8.7–28.98) capilia negative results among 60 culture positive samples which confirmed positive by MTBDR *plus* assay and PCR. Monde et al., 2013 reported 4/52 (7.69%) capilia negative isolates that were identified as MTBC by LPA [11]. In another report 6/247 (2.47%) isolates tested negative by capilia but positive by biochemical tests [12]. Reports from Japan suggested that 12/381 (3.15%) capilia TB negative isolates were positive by accuprobe and DNA-DNA hybridization test [13]. Gomathi et al., 2012 compared the results of capilia negative samples with conventional biochemical tests and found 66/114 (57.8%) as positive by conventional methods [14].

The discrepancies in the results of capilia were extensively evaluated by DNA sequencing of MPT64 region. In 7 positive cultures of our study stop codon occurred after 41st amino acid due to mutation and in 2 other positive cultures stop codon occurred at 38th and 88th amino acid. In 1 positive culture, insertion of G nucleotide caused a frame-shift mutation, while Hirano et al., 2004 reported the mutation in the *MPB64* gene due to the stop codon in 2 different strains at nucleotides 278 to 280 and 400 to 402 and in five strains observed the deletion of C-terminal 58 amino acids [13]. In a study by Ngamlert et al., 2009 mutations in the *MPB64* gene were detected in 5 isolates where 63 bp deletions at 196 bp and in one isolate 2 bp insertion at 436 bp [12]. Similarly, Ming-chih et al., 2011 study showed that 2 false negative isolates revealed a 63 bp deletion at position 196 in the *MPB64* gene in both isolates [15]. Hillemann et al., 2005 also reported mutation in *MPB64* gene, insertion of 1 bp created a frame-shift, nucleotide exchange resulting in a stop codon, and also disruption of the sequence due to an insertion of a copy of IS6110 [16]. Yan et al., 2015 reported mutation of 63-bp deletion and single base mutation in *MPT64* gene on MTB diagnosis [10]. Differences in finding of genetic alterations in this study as compared to other reports are possibly due to genotypic variation of strains.

Hence, our study indicates that three nucleotide positions viz., 3rd, 26th & 37th are very critical in the Indian scenario and have either insertion or deletion leading to frameshift. However, besides these three critical positions some additional mutations were also detected in the mutant positive cultures leading to further changes in the frame. All these mutations have lead to the expressions of a 41 amino acid long peptide in 70% of the mutant positive cultures which are entirely different from the wild type MPT64 protein. The remaining 30% of the mutant positive cultures had different phenotypic expression of the peptides due to additional mutation.

Thus, it is quite evident from our results that the mutant positive cultures with 41 amino acid long MPT64 protein in majority of cases had changes incorporated after 3rd, 9th & 13th amino acids due to frameshift which lead to no immuno-chromatographic identifications with monoclonal anti-MPT64 antibody of the capilia test.

One limitation of the study was that we did not perform conventional biochemical tests on culture and capilia positive isolates. Further identification of the MOTT strains is being carried out.

## Conclusions

In conclusion, genetic alteration in *MPT64* gene could be a major cause of false negative results of capilia test. Therefore, these mutations must be considered before implementing the antibody based capilia test for routine diagnosis as they can influence the test performance. Such cases can act as chronic carriers of TB in the population leading to rise of this lethal disease.

## Data Availability

The datasets generated and/or analyzed during the current study are not publicly available due confidentiality agreement at the department of microbiology, Institute of Medical Sciences, Banaras Hindu University but are available from the corresponding author on reasonable request.

## Abbreviations

BHI: Brain Heart Infusion agar
DST: Drug sensitivity testing
LPA: Line Probe Assay
MGIT: Mycobacterium Growth Indicator Tube
MOTT: Mycobacterium other than tuberculosis
MTB: *Mycobacterium tuberculosis*
MTBC: *Mycobacterium tuberculosis* complex
NALC-NaOH: N-acetyl-L-cysteine and sodium hydroxide
RIF&INH: Rifampicin & isoniazid
RNTCP: Revised National Tuberculosis Control Program
RR: Rifampicin resistance
RRDR: Rifampicin resistance determining region
WHO: World Health Organization
